# Brownian Motion Influence on AFM Exosomes’ Size Measurements

**DOI:** 10.3390/ijms231710074

**Published:** 2022-09-03

**Authors:** Katarzyna Życieńska, Beata Pszczółkowska, Beata Brzozowska, Maciej Kamiński, Tomasz Lorenc, Wioletta Olejarz, Sławomir Sęk, Józef Ginter

**Affiliations:** 1 Biomedical Physics Division, Institute of Experimental Physics, Faculty of Physics, University of Warsaw, 5 Pasteura Street, 02-093 Warsaw, Poland; 2 1st Department of Clinical Radiology, Medical University of Warsaw, 5 Chałubińskiego Street, 02-004 Warsaw, Poland; 3 Department of Biochemistry and Pharmacogenomics, Faculty of Pharmacy, Medical University of Warsaw, 1 Banacha Street, 02-097 Warsaw, Poland; 4 Centre for Preclinical Research, Medical University of Warsaw, 1b Banacha Street, 02-097 Warsaw, Poland; 5 Biological and Chemical Research Centre, Faculty of Chemistry, University of Warsaw, 101 Żwirki i Wigury Street, 02-089 Warsaw, Poland

**Keywords:** extracellular vesicles, exosomes, prostate cancer, atomic force microscopy (AFM), nanoparticle tracking analysis (NTA), Brownian motion

## Abstract

Extracellular vesicles are evaluated by nanoparticle tracking analysis (NTA), providing information on their hydrodynamic diameters, and by atomic force microscopy (AFM) to calculate their geometric diameters. The aim of this study is to explore the influence of Brownian movements in a sample drop and preparation time on imaging-based measurements and to determine the relationship between the geometric and hydrodynamic sizes of the extracellular vesicles measured by the AFM and the NTA, respectively. Exosomes derived from the human prostate cancer cell line PC3 were evaluated by NTA and AFM, and those results were compared with Monte Carlo simulations. The mean size, evaluated by AFM shortly after application on the mica substrate, is less than its real value. It obtains the correct value faster for a thinner sample drop. Fitting the log-normal distribution to the geometric and hydrodynamic diameters leads to the conclusion that the latter could arise from the former by linear scaling by a factor that could be used to characterize the analyzed extracellular vesicles. The size of the vesicles attached to the mica substrate depends on time. The effect of Brownian motion and stretch of the lipid bilayer should be considered in the context of exosome AFM studies.

## 1. Introduction

Exosomes are lipid-bilayer-bound nanoscale extracellular vesicles (EVs) that are released by both healthy and cancerous cells and have a natural ability to carry functional biomolecules. Accumulating evidence indicates that exosomes may play important roles in cell communication, angiogenesis, and carcinogenesis and are suitable for monitoring cancer progression and metastasis [1,2,3]. Exosomes are present in biological fluids and may be a source of clinically or biologically relevant information about malignant tumors, which refers to liquid biopsy, which is less invasive than traditional tumor biopsies. Multimodal exosome analysis may represent a noninvasive method for the diagnosis and the proper treatment selection [4]. The effect of membrane mechanics and physical properties of exosomes may influence further analysis [5]. The structural and mechanical properties of extracellular vesicles influence their behavior, such as their interactions with cells, and determine their geometry and shape that correspond to their penetration and compression potential [6]. Furthermore, due to their lipid and cell surface composition, engineered exosomes preloaded with therapeutic agents may improve pharmacokinetics and bioavailability and minimize adverse reactions [3]. Moreover, reengineered exosomes also have the potential for clinical use as personalized contrast media and theranostic for simultaneous radiological imaging and cell-free therapy [7]. Despite recent progress in studies of diagnostic and analytic technologies of exosomes, more research is needed to make exosomes valuable prognostic and diagnostic biomarkers and reliable vehicles for therapeutics delivery. Elasticity, stiffness, and shear of the extracellular vesicles are important for exosome engineering, for the integrity of the membrane after loading the encapsulated agents, for the consistency of the produced compound, and for the efficacy of exosome-based drugs in patients [8,9].

Experimental and theoretical studies in the past decade have provided important information on exosome size, electrostatic interactions, and mechanical properties of EVs such as membrane stiffness or bending modulus [10,11]. Currently, several techniques are employed to characterize extracellular vesicles, including atomic force microscopy (AFM) and nanoparticle tracking analysis (NTA), which provide complementary information, in accordance with the guidelines of the International Society for Extracellular Vesicles [12]. NTA is a valuable tool for assessing the randomly moving vesicles by Brownian movements in liquid, providing information on their so-called hydrodynamic diameters. AFM directly scans the surface of an immobilized vesicle for subsequent calculation of its geometric diameter, which the vesicle poses in liquid, before it is immobilized on freshly cleaved mica. If we consider a vesicle as a stiff and non-electrical sphere, its hydrodynamic and geometric diameters should be equal. In practice, hydrodynamic values are usually bigger than geometric dimensions of the vesicles [13,14]. The type of extracellular vesicle influences the relationship between their geometric and hydrodynamic diameters [15]. The relation between these diameters is still poorly understood, as we are lacking structural and mechanical insights into EV properties. In particular, vesicles Brownian movements in a specimen drop can cause AFM-imaging-based measurements to be dependent on a sample preparation time. The aim of this study is to explore that influence. At the same time, it tries to determine the relationship between the geometric sizes of the vesicles measured on the AFM and their hydrodynamic sizes provided by the NTA. The presented study provides suggestions on the methodology of the analysis of exosomes’ biophysical properties.

## 2. Results

### 2.1. Measurements of Vesicles’ Geometric Diameters

**Figure 1:**
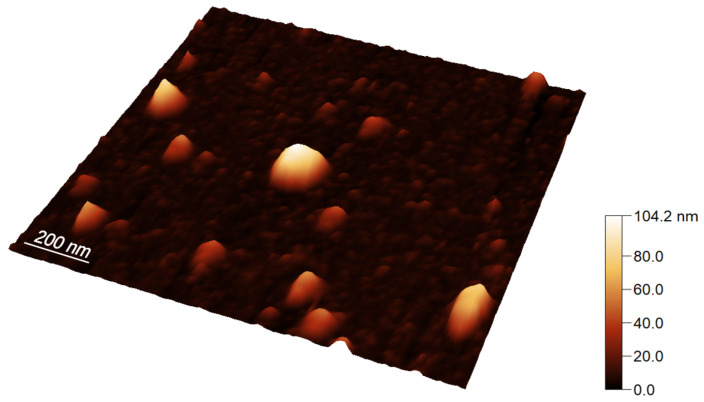
Three-dimensional representation of a mica fragment with EVs attached to it after 1 h from placing a drop of the sample, obtained from height imaging with AFM.

Figure 1 shows an exemplary mica-attached EVs landscape obtained from height imaging with AFM. One can see a large variation in the size of various vesicles with a height from a dozen to even 100 nanometers. Larger vesicles attract more attention, but numerically, the small and medium ones dominate. Some nano-objects appear to be elongated in shape. Perhaps this is just an artifact of the imaging method where it happens that the probe pulls the vesicle with it. This supposition would be confirmed by the direction of extension in line with the probe movement. Regardless of the reason of the elongation of such an object, only those with sufficient rotational symmetry can be treated as correctly imaged exosomes.

**Figure 2:**
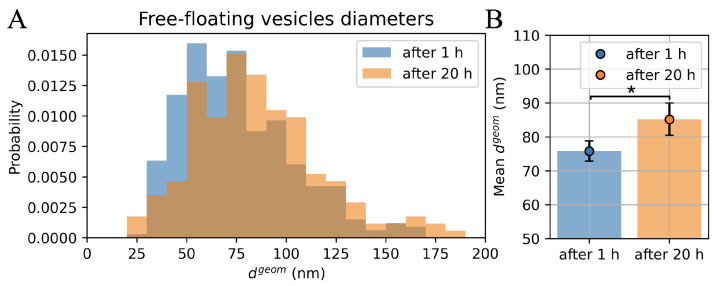
The exosome diameter as a function of imaging time: (**A**) histograms of the geometric diameters of exosomes isolated from PC3 cells, obtained on the basis of AFM images after 1 h (blue) and after 20 h (orange); (**B**) relevant mean values of vesicle diameters (blue and orange dots) together with a 95% CI obtained by bootstrapping; * means that 95% CI of the two outcomes do not overlap.

Figure 2A shows two distributions of vesicles’ diameters obtained using AFM imaging performed approximately 1 h and 20 h after the sample application. The histograms were made from 332 (13 images) and 172 (6 images) of the most symmetrical exosomes.

As can be seen, the histogram made after 20 h (marked in orange) is shifted to the right relative to the histogram of the vesicles diameters obtained after 1 h (shown in blue). This corresponds to the increase in the value of the mean diameter of the vesicles after a longer period of time, which is shown in Figure 2B by the dots of the corresponding colors. Error bars represent the 95% confidence interval (CI) obtained by bootstrapping. Mean geometric diameter with its CI after 1 h was 75.8 [−3.0, +3.1] nm, and after 20 h it was 85.2 [−4.7, +4.8] nm.

To explain the phenomenon of the shift to the right of the histogram of geometric diameters for the measurements after a longer period, two possible explanations have been proposed: (i) smaller vesicles move in the sample drop faster, so they reach the substrate earlier than larger ones; (ii) the vesicle lipid membrane slightly stretches over time.

### 2.2. Simulations of Brownian Movement in a Specimen Drop

A simulation was performed to estimate the influence of Brownian motion on the increase in the mean size of vesicles attached to the substrate surface over time. The hydrodynamic diameter of a given vesicle determines the speed of its random displacement in the liquid medium. The distribution of the hydrodynamic diameters in the sample can be obtained by NTA. These values are usually bigger than the geometric dimensions of the vesicles obtained by AFM imaging due to attractive forces with the surrounding water molecules [13] and also because the collisions of water molecules with the vesicle causing its movement are not entirely elastic [15]. While there is no universal relationship between the geometric diameter and the hydrodynamic diameter, it can be assumed that the bigger the first, the bigger the second as well. If this is the case, then the simulation of the attachment of vesicles on a mica substrate depending on their hydrodynamic diameter also qualitatively reflects their behavior depending on the geometric size.

**Figure 3:**
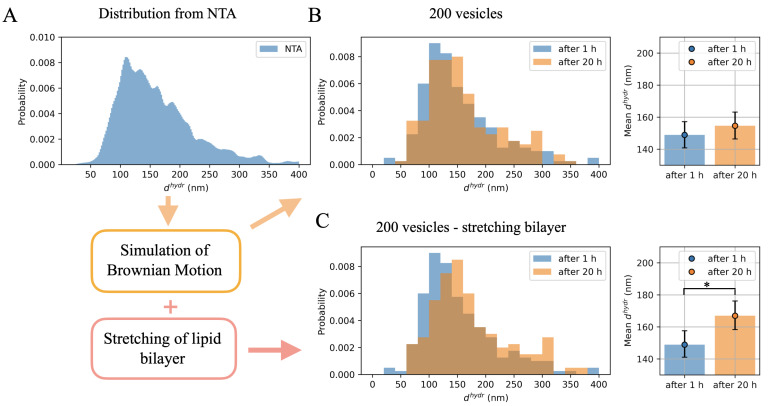
Results of the Monte Carlo simulation of adhesion of vesicles with hydrodynamic diameters drawn from the distribution obtained from NTA (**A**). In (**B**) the hydrodynamic diameter distributions of 200 vesicles are randomly selected out of those attached to the surface. The blue histogram corresponds to the situation after 1 h and the orange one after 20 h from placing a sample drop of 1 mm thick on the mica substrate. (**C**): taking into account the additional 8% linear membrane stretching for 200 vesicles selected after 20 h made the shift in time of mean vesicle hydrodynamic diameter statistically significant; * means that 95% CI of the two outcomes do not overlap.

Figure 3A shows the distribution of hydrodynamic diameters assumed in the simulation. In practice, the statistical significance of the effect of the mean vesicle size increase in time depends on how many vesicles have been analyzed. Figure 3B shows histograms for 200 vesicles randomly selected from those that settled on the substrate after 1 h (blue) and after 20 h (orange). On the right, the mean values of hydrodynamic vesicles diameters after shorter and longer periods, respectively, together with the 95% CI obtained by the bootstrap method, are shown as blue and orange dots. An increase in the mean value can be seen after 20 h, but it is not statistically significant.

Qualitatively, the obtained simulation results were similar to the results of the experiment; however, the increase in the mean value of vesicle size was slightly too small to obtain a statistically significant difference between the two simulations performed for 200 vesicles each. So what additional simulation condition should be met? In order to propose a possible answer to this question, the comparison of the average sizes of the 200 vesicles attached after 1 h and 20 h was also made assuming that after a longer time the lipid membrane was linearly stretched by 8%. In this case, the difference between the values of the mean hydrodynamic diameters of vesicles settled on mica after different time periods turns out to be statistically significant (see Figure 3C). The mean hydrodynamic diameter with its 95% CI after 1 h was 148.9 [−7.9, +8.6] nm and after 20 h it was 167.0 [−8.7, +9.2].

**Figure 4:**
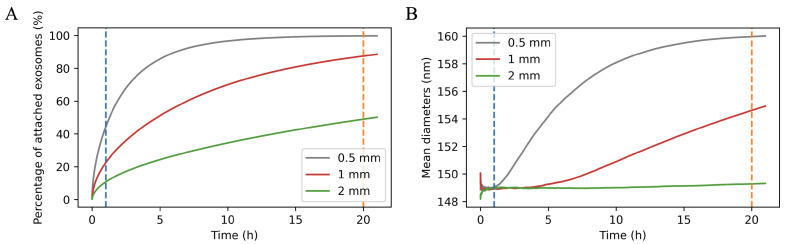
Characteristics of the nanovesicles attached to the substrate as a function of the time from applying the sample on mica, based on the Monte Carlo simulation: (**A**) percentage of attached vesicles; (**B**) mean hydrodynamic diameter of attached vesicles.

The percentage of vesicles attached to the substrate among all vesicles taken for the simulation increases over time (Figure 4A) as does the average value of their diameter (Figure 4B). These values were calculated for three different droplet thicknesses: 0.5 mm, 1 mm, and 2 mm. For a thickness of 1 mm (which best corresponded to the droplet thickness in the experiment), it can be seen that after 1 h about 20% of the vesicles were attached, while after 20 h more than 80% of them settled on a substrate.

### 2.3. Lognormal Distribution

The simulation described above showed the hydrodynamic diameters of the vesicles measured by the AFM. However, in reality, AFM does not provide hydrodynamic diameters, only geometric diameters. This section includes guidance on the relationship between the first and the second.

**Figure 5:**
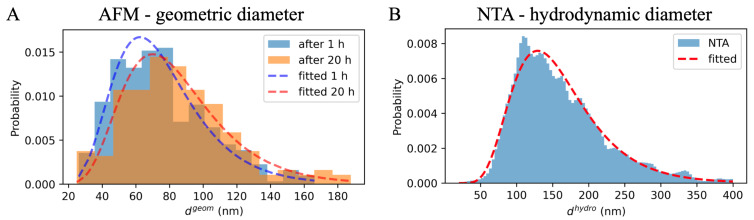
Log-normal distributions fitted to geometric diameter histograms from AFM (**A**) and to hydrodynamic diameter histograms from NTA (**B**).

The log-normal distribution was fitted to the geometric diameters measured with AFM after 1 h and 20 h, and to the sample of hydrodynamic diameters randomized from the distribution obtained from NTA. The sample size corresponded to the number of nano-objects included in the NTA measurement. As can be seen in Figure 5, the log-normal distribution’s shape fits well with the experimental data (certainly much better than the normal distribution could do). The values of fitted parameters $\mu^{*}$ and $\sigma^{*}$ are presented in Table 1. For the fit, the formulas presented by Lisicki et al. [16] were used; 95% CI was calculated using the bootstrapping method. $\mu^{*}$ is equal to the distribution’s median value, while $\sigma^{*}$ reflects its shape analogically to standard deviation for normal distribution, i.e., 68.3% of the histogram content is between $\mu^{*}/\sigma^{*}$ and $\mu^{*}\sigma^{*}$. Similar values of the shape parameter of various log-normal distributions might suggest that one can be obtained from another by linear scaling. More on the meaning of the parameters of the log-normal distribution can be found in [17].

The values of the $\sigma^{*}$ parameter for log-normal distributions fitted to geometric and hydrodynamic diameters are equal within confidence interval. This result suggests that the second of these distributions could arise from the first by linear scaling, i.e., multiplying geometric diameters of the vesicles by the $\mu_{NTA}^{*}/\mu_{AFM}^{*}$ ratio. Additional studies would be needed to check if this finding is general in nature or if it applies only to our exosome population.

In our case, it can be assumed that the hydrodynamic diameters of the vesicles were about twice their geometric diameters (149.2/71.1≈2.1). It can be seen that the simulation results described in Section 2.2 also indicated a difference in mean hydrodynamic diameters approximately twice as large as the difference in the mean value of geometric diameters after a short and long time, calculated on the basis of the experiment in Section 2.1. This would indicate the consistence of the proposed model of the process taking place in a sample drop placed on a mica substrate.

### 2.4. Hypothetical Bimodal Distribution

**Figure 6:**
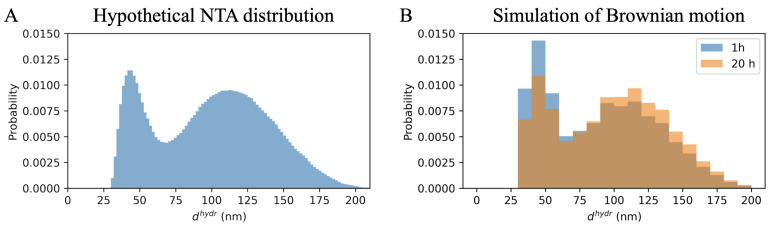
Results of the Monte Carlo simulation of settlement on the substrate of nanovesicles with hydrodynamic diameters drawn from the hypothetical bimodal distribution in (**A**). In (**B**), the blue histogram corresponds to 1 h, orange to 20 h from placing a 1 mm thick sample drop on the mica substrate.

The effect of Brownian motion in the sample droplet could have had an even bigger impact on the results of the AFM analysis if the vesicle size distribution were bimodal. Exosomes obtained from such a cell line have been studied in detail, for example in [18]. To check this effect, a simulation was performed for the hypothetical vesicles size distribution (Figure 6A). Figure 6B presents the size distributions of the vesicles attached to the substrate after 1 h (in blue) and after 20 h (in orange). It can be seen that, in such a situation, after a short time, small vesicles settled on the substrate surface would be strongly overrepresented in relation to the bigger ones.

## 3. Discussion

The effects of Brownian motion in the droplet of the test sample are usually overlooked in the context of exosome AFM studies. A typical procedure of exosome imaging involves performing a measurement several minutes after the sample application on the substrate (e.g., [19,20,21]). Nevertheless, it seems logical that bigger vesicles, having a lower diffusion constant, move slower in a liquid and therefore take longer to reach the bottom of the drop than smaller diameter vesicles. This relationship is noted by Skliar and Chernyshev [13] who suggest performing 12–18 h of EV incubation on the mica surface before AFM imaging.

Assuming that the hydrodynamic diameter is monotonically dependent on the geometric diameter (i.e., the bigger the geometric diameter, the bigger the hydrodynamic diameter), it can be concluded that both experiment and simulation reveal that the average size of the vesicles attached to the mica substrate depends on time.

The experimentally observed increase in the mean value of the geometric diameter by 10 nm was statistically significant when about 200 vesicles were analyzed. When simulating the settlement of a similar number of vesicles, for which, between 1 h and 20 h, the increase in the mean hydrodynamic diameter was about 6 nm, the result became statistically significant when the sample of approximately 2000 vesicles was simulated. In order to obtain statistical significance of the increase in the mean hydrodynamic diameter over time when modeling a sample of 200 vesicles, it was necessary to assume that their size after a longer period of time increased by an additional 8%, most probably due to stretching of the membrane.

Although the lipid bilayer is often considered inextensible in calculations, a slight stretching of the lipid film occurs [11,22]. In this experiment, this could be due to both the adhesion forces to the substrate and the hypotonic concentration of the environment. In both cases, an osmotic pressure is created between the inner and outer sides of the lipid membrane. The change in the surface area of the vesicles would be related to the penetration by osmosis of surrounding water into the vesicle, which may take time.

The obtained results do not prove that the typical procedure of AFM vesicles measurement shortly after sample placement needs to be changed. However, they indicate that caution should be taken when drawing conclusions from their results. For example, it seems that when such a procedure is used, it should be assumed that the actual mean vesicle size in the sample is several nanometers bigger than it seems from the image analysis. Within 1 h after placing the sample on the substrate, only some of the vesicles will attach, and it is not a representative sample.

Based on the presented results, one may conclude that samples should be analyzed after a few hours. However, such a conclusion is justified on the assumption that while waiting for the test, no additional effects causing an increase in the vesicle diameter take place. In the experiment discussed, this factor could be the hypotonic solution.

In the simulations described above, the influence of the droplet thickness on the result of the mean diameter value revealed that for a thinner droplet 90% of the vesicles would require less time to fall to the substrate. In this case, the total number of vesicles would be correspondingly smaller, but it turns out that when using the short waiting time for AFM imaging, applying a smaller amount of sample could give an even better result.

The median values of the fitted log-normal distributions differ significantly, but the values of the sigma parameter remain similar for all of them. This may mean that in practice hydrodynamic diameters arise from the multiplication of the geometric diameters by a constant factor. It can be expected that for different types of vesicles this factor will be different depending on quantities such as the stiffness [15] or the protein content of the lipid membrane [23]. It seems that the value of the ratio of the median of the hydrodynamic diameter distribution (with NTA) to the median of the geometric diameter distribution (with AFM) could be treated as an interesting physical parameter characterizing various types of vesicles. This would require confirmation in subsequent experiments.

## 4. Materials and Methods

### 4.1. Extracellular Vesicle Preparation

For the study, the human prostate cancer cell line PC3 (from the American Type Culture Collection, ATCC CRL-1435) was cultured in Dulbecco’s modified Eagle medium (DMEM-F12, Biowest, Nuaille, France, Cat # L0092-500) supplemented with 10% fetal bovine serum (FBS, Biowest, Cat. # S1810-500) and 2.0% penicillin-streptomycin (AppliChem GmbH, Darmstadt, Germany, Cat. # A8943). Cells were incubated at 37 °C in a humidified atmosphere containing 5% CO_2_. Cells were grown in T-75 flasks as a monolayer. Subculture was carried out routinely until the 75–80% cell confluence using 0.25% trypsin-EDTA (Biowest, Cat. # X0930-100) in a 1:5 ratio.

Six days before exosome isolation, cells were seeded into glass coverslips. The number of cells seeded on the single coverslips was $2.0\times 10^{5}$ cells/well. The coverslips with seeded cells were placed in a 6-well plate. Two days later, each glass was placed inside the Petri dish with fresh medium without FBS (only DMEM-F12 and 2% penicillin-streptomycin) and then placed in an incubator for four days. Afterwards, the culture supernatants were collected and used for exosome isolation. The isolation procedure included: purifying the supernatant by centrifugation twice under different conditions, then filtering through a microfilter (Millex 0.22 µm, EMD Millipore, Billerica, MA, USA, Cat. # SLGP033RS) to remove lasting cells, apoptotic bodies and bigger microvesicles. The next step was to concentrate the supernatant to 1 mL using the Vivacell 100 concentrator (100,000 MWCO, Sartorius, Stonehose, UK, Cat. # VS2041). The final step in the isolation was to filter the supernatant with a mini-SEC column (BioRad, Hercules, CA, USA, Cat. #7 321010) to remove bacteria, mitochondrial fragments, and extracellular vesicles bigger than exosomes, and to obtain the fraction with the most exosomes (fraction #4) [24]. The exosomes were stored at −80 °C until NTA or AFM analysis.

### 4.2. Nanoparticle Tracking Analysis (NTA)

The size and concentration of exosomes isolated from PC3 cells were studied using NTA analysis with the ZetaView device (Particle Metrix, Ammersee, Germany). Histograms of the number of EVs with different hydrodynamic diameters were obtained from three separate NTA measurements for the same type of EVs. Prior to measurement, the equipment was calibrated with 100 nm nano standard particles. The sample was diluted with PBS that had been previously checked using NTA for contamination. Between 700 and 1000 particles were tracked during the one measurement.

The presence of exosomes in the sample was confirmed by fluorescence measurement of NTA with CD9 antibodies (cfr. [25]).

### 4.3. Atomic Force Microscopy (AFM)

The distribution of geometric diameters of the exosomes was determined by AFM imaging (Dimension Icon-Bruker Instruments, Santa Barbara, CA, USA). The sample of a volume of 150 µL with the highest concentration of $3\times 10^{9}$ vesicles per mL was applied in the form of a drop on the surface of freshly cleaved mica disc with a diameter of 1 cm. The positive surface charge of the substrate was obtained by silanization of mica with 3-aminopropyltriethoxysilane (APTES) to achieve electrostatic attraction with vesicles having a negative surface charge, cfr. [26].

To ensure the cleanliness of the mica surface, its upper layer was first torn off. A drop of 30 µL of APTES solution in toluene 0.03% v/v was applied on it, and after 3 min, the mica was washed with ethyl alcohol and ultrapure water. Prior to a sample application, the cleanliness of the substrate was tested by AFM imaging in ultrapure water. The contamination level was required not to exceed 1 nano-object/µm^2^. For imaging, the mica was placed on a small stand with a slightly smaller diameter than the substrate in order to prevent any contamination from the plastic surface on which the test was carried out.

The AFM imaging was performed in PeakForce Tapping mode, with cantilever oscillating at the frequency of 2 kHz, much lower than its resonant frequency (cfr. [27]). The PEAKFORCE-HIRS-F-A probe with a radius of 1 nm was used. The probe pressure force was set automatically, limited to 600 pN to avoid damaging the vesicles.

The sample was examined twice. The first imaging started about 45 min after the sample was placed on the substrate. Subsequently, the mica, along with the drop of liquid, was placed in a closed plastic container with humid air at room temperature. The next imaging was performed on the next day, after about 20 h since the sample was set up. Respectively 13 and 6 images of an area of 3 × 3 µm^2^ were taken. During the imaging, a column of liquid was created between the sample and the probe by adding 100 µL of ultrapure water to the sample.

### 4.4. AFM Image Analysis

**Figure 7:**
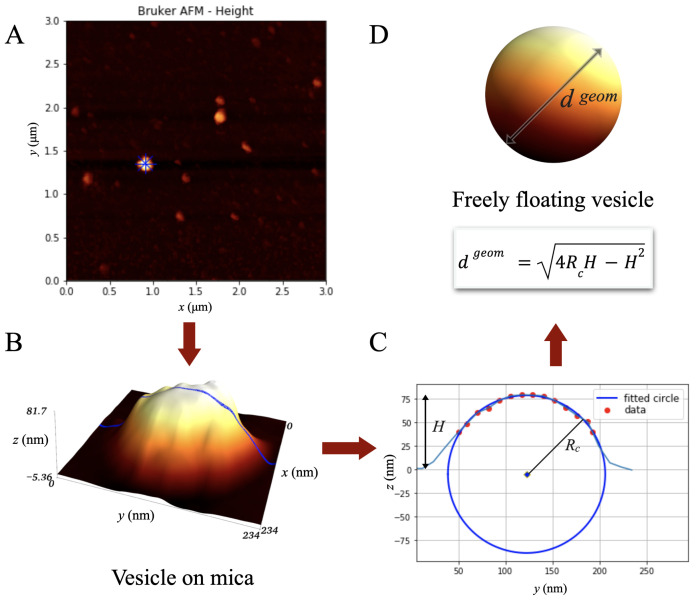
Scheme of AFM data analysis using AFM height mode images: (**A**) an exemplary AFM image (blue lines indicate directions of four profiles chosen for the selected vesicle); the height above the surface is coded with the heat colormap; (**B**) the selected exosome height is shown in 3D; one of the selected profiles is represented by the blue line; (**C**) the result of fitting a circle to the upper part of the selected profile; (**D**) evaluation and visualization of a freely floating vesicle with diameter $d^{geom}$ based on the fitted $R_{c}$ value.

AFM results were analyzed using custom written Python 3.7 scripts (we used the free Google Colaboratory Project https://colab.research.google.com/). Initial image processing was performed to remove scanning-related artifacts and set the zero level height to its mean value over the image. The algorithm detected 2D-Gaussian shaped blobs with a full width at half maximum (FWHM), describing the size of the blobs below 200 nm and a height bigger than 10 nm. The latter requirement was based on the assumption that the vesicles of interest are bounded by a double lipid membrane approximately 4–5 nm thick [28]. Next, four profiles were created for each of the blobs: along vertical and horizontal directions and along angles of 45 and 135 degrees (marked in blue Figure 7A). From these profiles, the four values of ${\mathrm{FWHM}}_{p}$ and the four heights $H_{p}$ were obtained. The selected exosome 3D representation is shown in Figure 7B. In order to extract the most circular-shaped objects, they were all arranged according to the asymmetry parameter described as $\frac{\mathrm{Max}\left({\mathrm{FWHM}}_{p}\right)-\mathrm{Min}\left({\mathrm{FWHM}}_{p}\right)}{\mathrm{Average}({\mathrm{FWHM}}_{p})}$. The histogram of this parameter had an inflection point for a value of about 0.6, which was treated as an arbitrary value above which the objects were rejected.

Then a circle was fitted to the measurement points on the upper part ($z>\frac{1}{2}H$) of each profile (Figure 7C). Assuming that the vesicles on the mica surface are shaped approximately as a spherical cap, the $R_{c}$ radius was calculated as the mean value of the radii of the fitted circles. The $R_{c}$ calculations allowed to estimate the diameter $d^{geom}$ of the free-floating vesicles in the liquid before their deposition on the substrate (Figure 7D). This can be performed [11] based on Formula (1):

(1) $$d^{geom}=\sqrt{4R_{c}H-H^{2}}.$$

The above formula assumes a constant value of the vesicle surface area, since the lipid bilayer does not stretch. This assumption is an approximation, more or less dependent on the protein molecules content of the membrane.

### 4.5. Simulations of Brownian Movement in a Specimen Drop

According to the Einstein–Stokes model [29,30], for an object with a hydrodynamic diameter $d^{hydr}$, the diffusion constant *D* is given by Formula (2):

(2) $$D=\frac{k_{B}T}{3\pi \eta d^{hydr}},$$

where $k_{B}$—Boltzmann constant, *T*—temperature, η—liquid viscosity. The diffusion constant tells how quickly nano-objects located initially all at one point would spread, namely, that the variance of the distribution of their positions along the chosen direction (let us name it *z*-axis) would increase linearly with time: $\langle z^{2}\rangle =2Dt$.

The behavior of the vesicles was simulated using the Monte Carlo method by custom written Python 3.7 scripts. First, random positions of $N=2\times 10^{6}$ vesicles were determined along the z-axis (height above the substrate), randomized from a homogeneous distribution in the range (0, $z_{max}$), where $z_{max}$ is the drop thickness. For the *k*-th vesicle, the hydrodynamic diameter $d_{k}^{hydr}$ was drawn from the probability distribution obtained on the basis of the average size histogram from three separate NTA measurements for the same type of EVs. In subsequent moments, the positions $z_{k}$ changed their values by adding to them random values from the Gaussian distribution with standard deviation $\sigma_{k}=2D_{k}\Delta t$, where the diffusion constants $D_{k}$ were calculated on the basis of their $d_{k}^{hydr}$ values in accordance with Formula (2), and Δt is the time step. The assumption was that a vesicle that touches the mica surface remains attached to it and is removed from further simulation. On the other hand, when a vesicle touches the upper border of the drop, it bounces off it.

In the calculations, the drop was treated as if it was a cylinder, without taking into account its spherical shape. However, the height of the cylinder was calculated as the average height of the points on the drop’s spherical cap with a base radius $r_{base}$ = 5 mm (mica size) and height $h_{drop}$ = 1 mm. It was calculated from Formula (3):

(3) $$h_{cylinder}=\frac{1}{2}h_{drop}+\frac{1}{6}\frac{h_{drop}^{3}}{r_{base}^{2}}$$

The simulation was carried out for the time from 0 to 20 h with the time step equal to 10 s.

Additionally characteristics of the nanovesicles attached to the substrate as a function of the time (percentage of attached vesicles and mean hydrodynamic diameter) were compared for three drop’s heights: 0.5 mm, 1 mm and 2 mm on the base of $1\times 10^{6}$, $2\times 10^{6}$ and $4\times 10^{6}$ vesicles respectively. The use of different numbers was supposed to represent the same vesicles concentration in different drop volumes.

## 5. Conclusions

Two main methodological conclusions emerge from our research:

(i) A reasonable compromise should be made between droplet thickness and waiting time before AFM imaging. Based on the results presented in Figure 3, it can be assumed that a drop 0.5 mm thick (with sufficient vesicles concentration) and time before imaging 5–10 h (with adequate humidity so that the sample would not dry during this time) will allow obtaining a representative statistical sample of vesicles attached to the mica substrate.
(ii) It is suggested as an additional method of characterizing the physical properties of EVs to find the ratio of the hydrodynamic diameter to the geometric diameter. For this purpose, on the basis of AFM imaging, a histogram of geometric diameters of the vesicles should be created (see Section 4.4), and a histogram of the hydrodynamic diameters should be obtained using NTA. If fitting the log-normal distributions to this data, you obtain $\sigma_{NTA}^{*}\approx \sigma_{AFM}^{*}$, then the quantity you are looking for will be the ratio $\mu_{NTA}^{*}/\mu_{AFM}^{*}$.

## Figures and Tables

**Table 1:** Parameters of log-normal distribution fitted to geometric and hydrodynamic diameters with its 95% CI estimated from bootstrap.

	Scale $\mu^{*}$ (nm)	Shape $\sigma^{*}$ (nm)
geometric diameters AFM after 1 h	71.1 [−2.8, +3.0]	1.431 [−0.035, +0.033]
geometric diameters AFM after 20 h	79.8 [−4.5, +4.7]	1.436 [−0.054, +0.050]
hydrodynamic diameters NTA	149.2 [−2.3, +2.3]	1.461 [−0.014, +0.014]

## Data Availability

Analysed data and Python scripts can be found available at https://github.com/zycienska/AFM_BrownianMotion.

## Abbreviations

The following abbreviations are used in this manuscript:

EVs	extracelular vesicles
AFM	Atomic Force Microscopy
NTA	Nanoparticle Tracking Analysis
FBS	fetal bovine serum
APTES	3-aminopropyltriethoxysilane
FWHM	full width at half maximum
CI	confidence interval
